# A Fast Smoothing Algorithm for Post-Processing of Surface Reflectance Spectra Retrieved from Airborne Imaging Spectrometer Data

**DOI:** 10.3390/s131013879

**Published:** 2013-10-14

**Authors:** Bo-Cai Gao, Ming Liu

**Affiliations:** 1 Remote Sensing Division, Code 7232, Naval Research Laboratory, Washington, DC 20375, USA; 2 Software Branch, Field System Operation Center, NOAA, Silver Spring, MD 20910, USA; E-Mail: ming.liu@noaa.gov

**Keywords:** remote sensing, sensors, spectroscopy, smoothing, surface reflectance

## Abstract

Surface reflectance spectra retrieved from remotely sensed hyperspectral imaging data using radiative transfer models often contain residual atmospheric absorption and scattering effects. The reflectance spectra may also contain minor artifacts due to errors in radiometric and spectral calibrations. We have developed a fast smoothing technique for post-processing of retrieved surface reflectance spectra. In the present spectral smoothing technique, model-derived reflectance spectra are first fit using moving filters derived with a cubic spline smoothing algorithm. A common gain curve, which contains minor artifacts in the model-derived reflectance spectra, is then derived. This gain curve is finally applied to all of the reflectance spectra in a scene to obtain the spectrally smoothed surface reflectance spectra. Results from analysis of hyperspectral imaging data collected with the Airborne Visible/Infrared Imaging Spectrometer (AVIRIS) data are given. Comparisons between the smoothed spectra and those derived with the empirical line method are also presented.

## 1. Introduction

Since the mid-1980s, hyperspectral imaging data have been collected with different types of imaging spectrometers from aircraft and satellite platforms. Because solar radiation along the sun-surface-sensor path in the 0.4−2.5 μm visible and near-IR spectral regions is subject to absorption and scattering by atmospheric gases and aerosols, hyperspectral imaging data contain atmospheric effects. In order to use hyperspectral imaging data for quantitative remote sensing of land surfaces and ocean color, the atmospheric effects must be removed. There are now a number of model-based atmospheric correction algorithms for retrieving surface reflectances from hyperspectral imaging data. These include, but are not limited to, the ATmosphere REMoval algorithm (ATREM) [1,2], the High-accuracy ATmospheric Correction for Hyperspectral Data (HATCH) [3], the Atmosphere CORrection Now (ACORN) [4], the Fast Line-of-sight Atmospheric Analysis of Spectral Hypercubes (FLAASH) [5], the Imaging Spectrometer Data Analysis System (ISDAS) [6], and a series of Atmospheric and Topographic Correction (ATCOR) codes [7,8].

**Figure 1.**
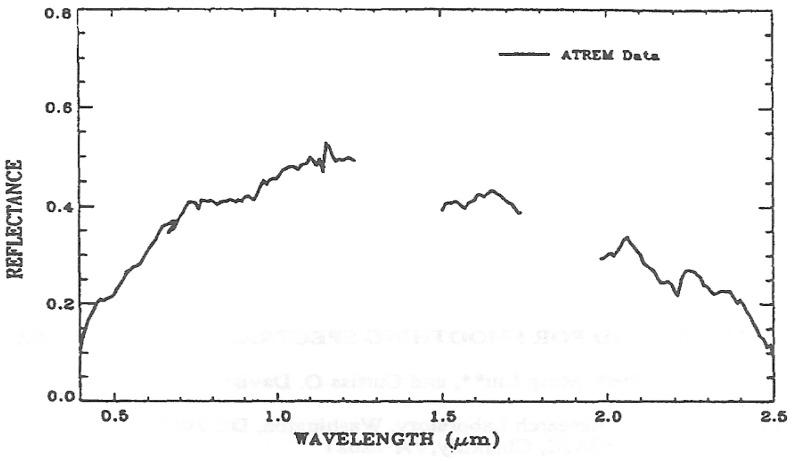
An example of a reflectance spectrum derived with ATREM from AVIRIS data acquired in June, 1995 over Cuprite, Nevada, USA.

The surface reflectance spectra retrieved with radiative transfer models often contain residual atmospheric absorption and scattering effects. The reflectance spectra can also contain artifacts due to errors in radiometric and spectral calibrations. Although models have been improving with time, they are not yet at the level where all artifacts are smaller than sensor noise. Figure 1 shows an example of a reflectance spectrum derived with ATREM from AVIRIS data [9] acquired over Cuprite (NV, USA) in June, 1995. Due to small errors in assumed wavelengths and errors in line parameters compiled on the HITRAN database [10], small spikes (particularly near the centers of the 0.94- and 1.14-μm water vapor bands) are present in this spectrum. These spikes have distracted geologists who are interested in studying surface mineral features. Clark *et al.* [11] pioneered a hybrid approach for the derivation of laboratory-quality surface reflectance spectra from AVIRIS data. They used a combination of ATREM and field spectral measurements over a single ground calibration site. In this case the use of ATREM allows improved atmospheric corrections at elevations that are different from the calibration site, and the ground calibration removes residual errors commonly associated with sensor artifacts and radiative transfer models. In many situations, researchers do not have any field-measured reflectance spectra for suppressing residual errors.

Boardman [12] first developed the Empirical Flat Field Optimal Reflectance Transformation (EFFORT) method to suppress residual errors in ATREM-derived surface reflectance spectra without the need for field-measured reflectance spectra. In this method, the complete spectrum for each pixel in the 0.4–2.5 μm range is fitted with a low order polynomial. It is sometimes difficult to make reasonable matches over the entire (or “global”) spectral range. In this article, we describe another technique, which fits spectra “locally” in the spectral domain using moving filters derived with a cubic spline smoothing algorithm, for quick post processing of ATREM-derived reflectance spectra from imaging spectrometer data. Results from analysis of AVIRIS data acquired over the Cuprite mining district in Nevada in June of 1995 and over Ivanpah in California in April of 2010 are given. Comparisons between the smoothed spectra and those derived with the empirical line method are also presented.

## 2. Methodology

In order to describe the smoothing technique, we first describe the commonly used cubic spline “fitting” technique, then we describe the cubic spline “smoothing” technique.

### 2.1. Cubic Spline Fitting

**Figure 2.**
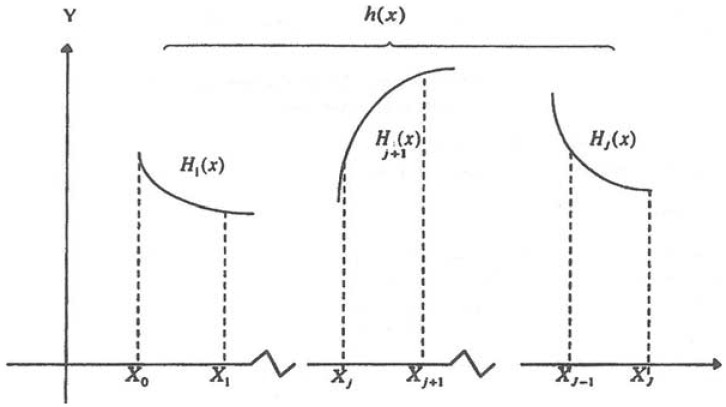
An illustration of the interpolating function *h(x)*.

The cubic spline fitting technique is a powerful numerical method and has been widely used in engineering and scientific computing. For example, Numerical Recipes [13] provides standard subroutines, using cubic spline fitting method, for interpolating data between points. In order to describe mathematically the cubic spline fitting technique, we consider an interval *α* ≤ *x* ≤ *b*, and subdivide it by a mesh of points corresponding to the location of the data at *α* = *X*_0_ < *X*_1_ <…< *X_j_*_−1_ < *X_j_*…< *X_J_* = *b*. An associated set of the observed data is prescribed by *y*_0_,*y*_1_,…,*y_j_*,…,*y_J_*. We seek an interpolating function *h(x)*, which is defined in the interval [a,b]. Its first and second derivatives are continuous on [a,b] and it coincides with a cubic polynomial in each subinterval *X_j_*_−1_ ≤ *x* ≤ *X_j_*, and satisfies the relationship *h_j_* = *h*(*X_j_*)=*y_j_*. Figure 2 illustrates the function *h(x)*. As adapted from Ahlberg [14], the function *h(x)* in the interval *X_j_*_−1_ ≤ *x* ≤ *X_j_* can be expressed as (for convenience, we assume the problem of equally spaced samples with a step size of Δ):

(1) $$h(x)=\{\begin{array}{cc} H_{1}(x) & X_{0}\leq x\leq X_{1}\\ : & :\\ H_{j}(x) & X_{j-1}\leq x\leq X_{j}\\ : & :\\ H_{J}(x) & X_{J-1}\leq x\leq X_{J} \end{array}$$

where:

(2) $$\begin{array}{l} H_{j}(x)=s_{j-1}\frac{{\left(X_{j}-x\right)}^{3}}{\Delta }+s_{j}\frac{{\left(x-X_{j-1}\right)}^{3}}{\Delta }+\left[h_{j-1}-s_{j-1}\right]\frac{\left(X_{j}-x\right)}{\Delta }+\left[h_{j}-s_{j}\right]\frac{\left(x-X_{j-1}\right)}{\Delta }\\ X_{j}=X_{0}+j\Delta \\ h_{j}=h(X_{j});j=0,1,2,‥J \end{array}$$

{*s_j_*}, the spline coefficients, can be interpreted as the normalized second derivatives.

The polynomials (2) in adjacent segments are continuous at the knots:

(3) $$H_{j}(X_{j})=h_{j}=H_{j+1}(X_{j})$$

The first derivative is continuous at the knot provided that:

(4) $$s_{j-1}+4s_{j}+s_{j+1}=h_{j-1}-2h_{j}+h_{j+1}$$

The second derivative is continuous at the knots:

(5) $$H_{j}^{"}(X_{j})=H_{j+1}^{"}(X_{j})=\frac{6}{\Delta^{2}}s_{j}$$

The polynomials (2) are determined by specification of {*s_j_*}. The selection of these spline coefficients can involve any number of imposed weak constraints that characterize the spline fitting. One of the constraints is the minimization of the second derivative. Because:

(6) $${\int_{X_{j-1}}^{X_{j}}\left[H^{"}(x)\right]}^{2}dx=\frac{12}{\Delta^{3}}\left[s_{j-1}^{2}+s_{j}^{2}+s_{j-1}s_{j}\right]$$

it follows that the quantity $\left[s_{j-1}^{2}+s_{j}^{2}+s_{j-1}s_{j}\right]$is to be minimized in any kind of variational selections of {*s_j_*}. The simplest quadratic form to minimize is $\sum_{j=1}^{J}\left[s_{j-1}^{2}+s_{j}^{2}+s_{j-1}s_{j}\right]$. However, this is not enough to guarantee continuity of the derivatives at the knots. A method to incorporate the condition:

(7) $$s_{j-1}+4s_{j}+4s_{j+1}-\left(h_{j-1}-2h_{j}+h_{j+1}\right)=0$$

must be found. This is done by introducing Lagrangian multipliers. The simple spline formulation for the minimization is:

(8) $$E\left[\left\{s_{j}\right\}\right]=\sum_{j=1}^{J}\left[s_{j-1}^{2}+s_{j}^{2}+s_{j-1}s_{j}\right]+2\sum_{j=1}^{J-1}\lambda_{j}\left[s_{j-1}+4s_{j}+s_{j+1}-\left(h_{j-1}-2h_{j}+h_{j+1}\right)\right]$$

where *λ_j_*s are the Lagrangian multipliers. These conditions are exactly satisfied upon completion of the minimization so that zeros are in effect added to the quantity to be minimized. The procedure of solving those *λ_j_*s, therefore, the spline coefficients {*s_j_*}, and the interpolating splines {*h_j_*}, is similar to that of spline smoothing to be described in the next section.

### 2.2. Cubic Spline Smoothing

In the spline fitting technique described above, the {*h_j_*} are taken to represent errorless data or observations, and the spline passes each point *y_j_*. However, there can be circumstances that the observations are contaminated and unwanted noise is present. For example, in our case raw spectra exhibit coherent saw-tooth like “noise”. Under these circumstances, the data integrity condition should be relaxed. This can be done by adding a weak constraint term, $\sum_{j=0}^{J}{\left[h_{j}-y_{j}\right]}^{2}$ to Equation (8), where *y_j_* is the observed data, and only a “best fit” should be sought. The smoothed spline {*h_j_*} does not necessarily pass original observed data {*y_j_*}, unlike the case of the spline fitting. An appropriate discrepancy sum can be formed as:

(9) $$E\left[\left\{s_{j}\},\{h_{j}\right\}\right]=\tau^{2}\sum_{j=1}^{J}\left[s_{j-1}^{2}+s_{j}^{2}+s_{j-1}s_{j}\right]+\sum_{j=0}^{J}{\left[h_{j}-y_{j}\right]}^{2}+2\sum_{j=1}^{J-1}\lambda_{j}\left[s_{j-1}+4s_{j}+s_{j+1}-\left(h_{j-1}-2h_{j}+h_{j+1}\right)\right]$$

where *τ*^2^ is an adjustable weighting factor. As it increases, the tension of the spline smoothing increases, *i.e.*, the curve “flattens out”. On the other hand, as it decreases, the observed data are reproduced more closely at the expense of increased curvature.

The variations on the spline coefficients are tabulated as:

(10) $$\begin{array}{cccc} \delta s_{0} & \tau^{2}\left(2s_{0}+s_{1}\right)+2\lambda & =0 & \\ \delta s_{1} & \tau^{2}\left(s_{0}+4s_{1}+s_{2}\right)+2\lambda_{1}+8\lambda_{2}+2\lambda_{3} & =0 & \\ : & : & : & \\ \delta s_{j} & \tau^{2}\left(s_{j-1}+4s_{j}+s_{j+1}\right)+2\lambda_{j-1}+8\lambda_{j}+2\lambda_{j+1} & =0 & j=2,\ldots ,(J-2)\\ : & : & : & \\ \delta s_{J-1} & \tau^{2}\left(s_{J-2}+4s_{J-1}+s_{J}\right)+2\lambda_{J-2}+8\lambda_{J-1} & =0 & \\ \delta s_{J} & \tau^{2}\left(s_{J-1}+s_{J}\right)+2\lambda_{J-1} & =0 & \end{array}$$

The variations on the multipliers lead to:

(11) $$\begin{array}{ccc} \delta \lambda_{j}: & s_{j-1}+4s_{j}+s_{j+1}=h_{j-1}-2h_{j}+h_{j+1}; & j=1,(J-1) \end{array}$$

Since the spline does not pass the data {*y_j_*}, the {*h_j_*} are no longer fixed; their variations are listed below:

(12) $$\begin{array}{cccc} \delta h_{0} & 2\left(h_{0}-y_{0}\right)-2\lambda_{1} & =0 & \\ \delta h_{1} & 2\left(h_{1}-y_{1}\right)+4\lambda_{1}-2\lambda_{2} & =0 & \\ : & : & : & \\ \delta h_{j} & 2\left(h_{j}-y_{j}\right)-2\lambda_{j-1}+4\lambda_{j}-2\lambda_{j+1} & =0 & j=2,\ldots ,(J-2)\\ : & : & : & \\ \delta h_{J-1} & 2\left(h_{J-1}-y_{J-1}\right)-2\lambda_{J-2}+4\lambda_{J-1} & =0 & \\ \delta h_{J} & 2\left(h_{J}-y_{J}\right)-2\lambda_{J-1} & =0 & \end{array}$$

Combining terms in (12), we have:

(13) $$\left[h_{j-1}-2h_{j}+h_{j+1}\right]-\left[y_{j-1}-2y_{j}+y_{j+1}\right]=\lambda_{j-2}-4\lambda_{j-1}+6\lambda_{j}-4\lambda_{j+1}+\lambda_{j+2}$$

Each of the combinations [*s_j_*_−1_ + 4*s_j_* + *s_j_*_+1_]; *j*=1,…,(*J*−1) in (10) can be replaced by their equivalents from (11) to obtain the following equations:

(14) $$\begin{array}{ccc} \delta s_{1}: & \tau^{2}\left(h_{0}-2h_{1}-h_{2}\right)+8\lambda_{1}+2\lambda_{2} & =0\\ \delta s_{j}: & \tau^{2}\left(h_{j-1}-2h_{j}-h_{j+1}\right)+2\lambda_{j-1}+8\lambda_{j}+2\lambda_{j+1} & =0\\ \delta s_{J-1}: & \tau^{2}\left(h_{0}-2h_{1}-h_{2}\right)+2\lambda_{J-2}+8\lambda_{J-1} & =0 \end{array}$$

The [*h_j_*_−1_−2*h_j_*+*h_j_*_+1_] in (14) can be replaced by the groupings in (13):

(15) $$\begin{array}{l} \begin{array}{ccc} \delta s_{1} & \tau^{2}\left[\Delta^{2}{ℵ}^{2}y_{1}+\left(6\lambda_{1}-4\lambda_{2}+\lambda_{3}\right)\right]+8\lambda_{1}+2\lambda_{2} & =0\\ \delta s_{2} & \tau^{2}\left[\Delta^{2}{ℵ}^{2}y_{2}+\left(-4\lambda_{1}+6\lambda_{2}-4\lambda_{3}+\lambda_{4}\right)\right]+2\lambda_{1}+8\lambda_{2}+2\lambda_{3} & =0\\ : & : & :\\ \delta s_{j} & \tau^{2}\left[\Delta^{2}{ℵ}^{2}y_{j}+\left(\lambda_{j-2}-4\lambda_{j-1}+6\lambda_{j}-4\lambda_{j+1}+\lambda_{j+2}\right)\right]+2\lambda_{j-1}+8\lambda_{j}+2\lambda_{j+1} & =0\\ : & : & :\\ \delta s_{J-2} & \tau^{2}\left[\Delta^{2}{ℵ}^{2}y_{J-2}+\left(\lambda_{J-4}-4\lambda_{J-3}+6\lambda_{J-2}-4\lambda_{J-1}\right)\right]+2\lambda_{J-3}+8\lambda_{J-2}+2\lambda_{J-1} & =0\\ \delta s_{J-1} & \tau^{2}\left[\Delta^{2}{ℵ}^{2}y_{J-1}+\left(6\lambda_{J-3}-4\lambda_{J-2}+\lambda_{J-1}\right)\right]+2\lambda_{J-2}+8\lambda_{J-1} & =0 \end{array}\;j=3,\ldots ,(J-3) \end{array}$$

where: ${ℵ}^{2}y_{j}=\frac{y_{j-1}-2y_{j}+y_{j+1}}{\Delta^{2}}$

The {*λ_j_*} are then found as solutions of:

(16) Aλ=y"

where **A** is the pentadiagonal matrix

(17) $$\left(\begin{array}{ccccccccccc} c & b & a & & & & & & & & \\ b & c & b & a & & & & & & & \\ a & b & c & b & a & & & & & & \\ & a & b & c & b & a & & & & & \\ & & a & b & c & b & a & & & & \\ & & & . & . & . & . & . & & & \\ & & & & . & . & . & . & . & & \\ & & & & & . & . & . & . & . & \\ & & & & & & a & b & c & b & a\\ & & & & & & & a & b & c & b\\ & & & & & & & & c & b & c \end{array}\right)$$

Here the matrix elements are constants and given by:

(18) $$\begin{array}{ccc} a & = & \tau^{2}\\ b & = & \left(2-4\tau^{2}\right)\\ c & = & \left(8+6\tau^{2}\right) \end{array}$$

and:

(19) $$\begin{array}{ccc} y" & = & -\Delta^{2}\tau^{2}\left(\begin{array}{c} {ℵ}^{2}y_{1}\\ {ℵ}^{2}y_{2}\\ .\\ {ℵ}^{2}y_{j}\\ .\\ {ℵ}^{2}y_{J-1} \end{array}\right) \end{array}$$

Given the {*λ_j_*}, the {*h_j_*} can be determined directly from (13), and the spline coefficients {*s_j_*} can be found as a tridiagonal solution of (10).

## 3. Procedures for Post Processing of ATREM-Derived Reflectance Data

**Figure 3.**
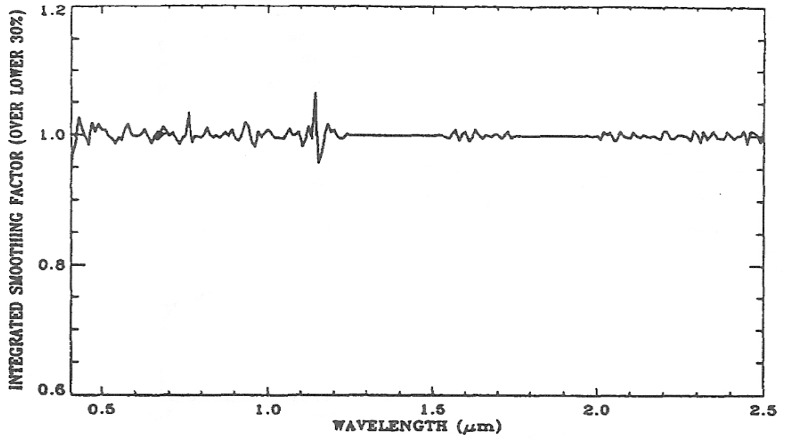
A sample gain spectrum.

The small spikes, as seen in Figure 1, are systematically present in all spectra in an ATREM output data cube (2-d spatial plus 1-d spectral). We hope to make mild “gain” adjustments to remove these small spikes during the post processing of the ATREM data cube. Specifically, we hope to find a gain function, g(*λ*), which contains all the weak spikes and which has a mean value close to 1. The multiplication of g(*λ*) to the ATREM output spectra should allow the removal of the systematic small spikes. Several steps are involved in the post processing of an ATREM output data cube. They are:

(a) The cubic spline smoothing technique described in Section 2 is applied to each of the spectra in the ATREM data cube. As a result, an intermediate “smoothed” data cube is produced. Because the cubic spline smoothing technique fits a spectrum “locally” in the spectral domain, most of the smoothed spectra at this stage match quite well with the ATREM spectra. If the spectra were fit with low order Legendre polynomials “globally”, only a minor fraction of the smoothed spectra would match well with the ATREM spectra.
(b) The average reflectance, *ρ*_avg_, is calculated for each of the spectra in the ATREM output data cube.
(c) For each pixel, the standard deviation, *σ*, between the ATREM spectrum and the “smoothed” spectrum is calculated.
(d) For an AVIRIS scene, a scatter plot of *σ*/*ρ*_avg_*vs. ρ*_avg_ is made. Pixels with *σ*/*ρ*_avg_ values in the lower twenty percentile are identified.
(e) For each of the pixels identified in Step d, a ratio spectrum (“smoothed” spectrum/ATREM spectrum) is calculated. The desired gain spectrum, g(*λ*), is obtained by averaging all the ratio spectra. Figure 3 shows an example of a gain spectrum, which contains a number of weak spikes in the 0.4–2.5 μm spectral region.
(f) Each of the spectra in the ATREM output data cube is multiplied by the gain spectrum to obtain the “final” smoothed data cube.

Our algorithm for smoothing the ATREM output data cube is fast. It takes approximately 30 s on a Mac computer with a 2.66 GHz Quad-Core Intel Xeon processor to process one complete data cube with a dimension of 614 samples, 972 lines, and 224 bands.

## 4. Sample Results

The cubic spline smoothing algorithm described above was implemented on an AVIRIS server computer at the NASA Jet Propulsion Laboratory for post-processing large volumes of ATREM-derived reflectance data cubes from AVIRIS radiances acquired during major field experiments. Below we present results from one set of AVIRIS data acquired over the Cuprite Mining District in Nevada in June, 1995 and another over Ivanpah playa in California in April, 2010.

### 4.1. Cuprite, Nevada

**Figure 4.**
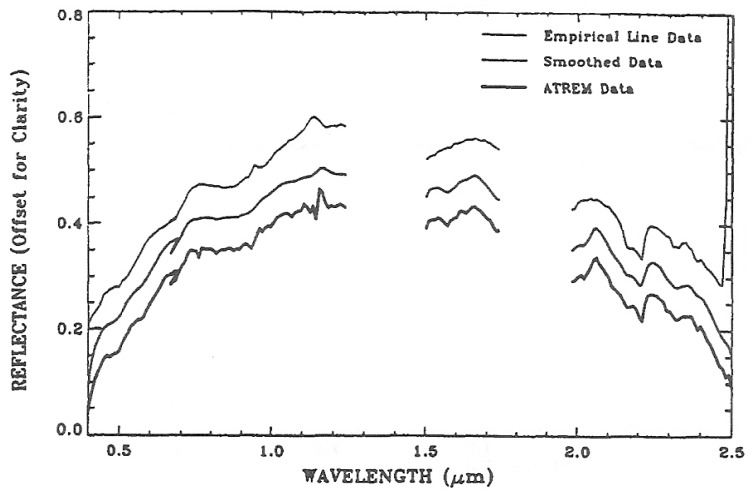
An ATREM reflectance spectrum (lower line), a smoothed spectrum (middle line), and a reflectance spectrum obtained with the empirical line method (upper line). For clarity, the three spectra are vertically offset.

Figure 4 shows a comparison among an ATREM reflectance spectrum (lower line) over a single pixel within the 1995 AVIRIS Cuprite scene, the smoothed spectrum (middle line), and the reflectance spectrum obtained with the well-known empirical line method (upper curve) [15]. For clarity, the spectra in Figure 4 are vertically displaced. The general shapes of these spectra in the 0.4–1.26 μm, 1.5–1.75 μm, and 2.0–2.5 μm wavelength intervals are very similar. Major mineral features in the 2.0–2.5 μm region are seen in all the spectra. The un-smoothed ATREM spectrum has quite a few weak spikes. These spikes are largely removed in the smoothed spectrum. The spectrum derived with the empirical line method shows weak inverse water vapor features near 0.94 and 1.14 μm. This indicates that the method results in a slight over-correction of atmospheric water vapor absorption effects for this pixel.

**Figure 5.**
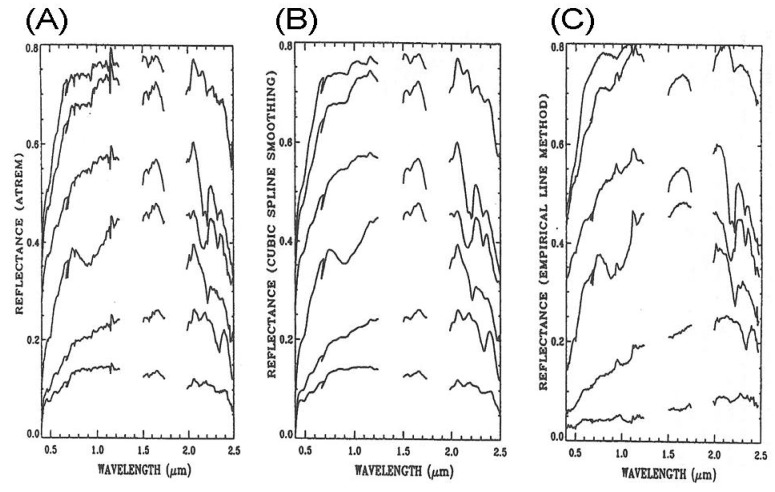
(**A**): Six reflectance spectra (displaced vertically for clarity) derived with ATREM from AVIRIS data acquired over Cuprite, Nevada in June, 1995; (**B**): six smoothed reflectance spectra corresponding to those in (A); and (**C**): six reflectance spectra derived with the empirical line method and corresponding to those in (A).

Figure 5A shows six ATREM reflectance spectra (vertically displaced for clarity). These spectra have distinct mineral absorption features in the 2.0–2.5 μm spectral region. Weak spikes (for example near 1.14 μm) are systematically present in all the spectra. Figure 5B shows the corresponding smoothed spectra, which look very similar to laboratory-measured reflectance spectra, particularly in the 2.0–2.5 μm spectral region. Weak spikes are all removed. A broad iron feature near 0.9 μm is seen nicely in one spectrum—the 4th spectrum from top. Figure 5C shows six spectra derived from the AVIRIS data with the empirical line method. Mineral features in the 2.0–2.5 μm region are recovered quite well with this method. However, water vapor features in the 0.94 and 1.14 μm regions are either over- or under- corrected. The broad iron feature in the 4th spectrum from the top is not clearly seen due to the over-correction of atmospheric water vapor absorption effects. By comparing Figured 5A–C, it is seen that major mineral features are preserved after the spectral smoothing.

### 4.2. Ivanpah, California

**Figure 6.**
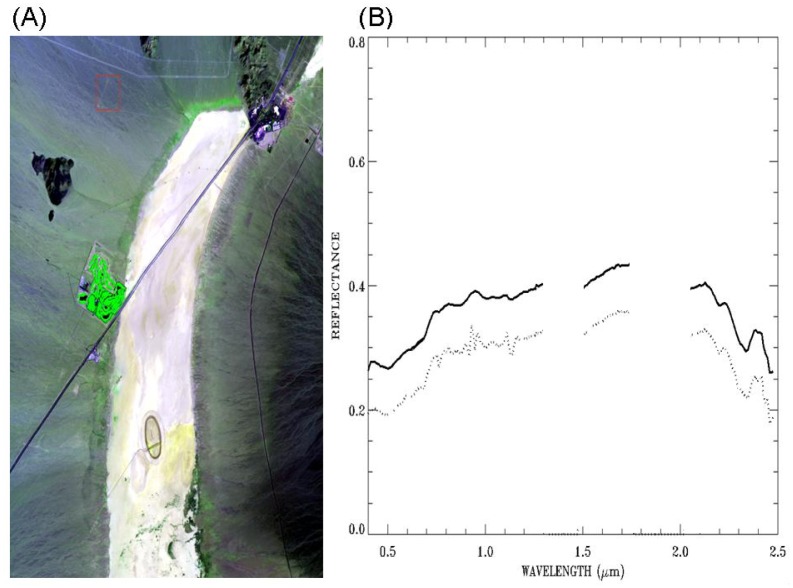
(**A**): a false color image (Red: 0.63 μm; Green: 0.86 μm; Blue: 0.47 μm) for an AVIRIS scene over Ivanpah in California, and (**B**): an ATREM-derived surface reflectance spectrum over a soil pixel (dotted line) and the corresponding smoothed spectrum (solid line). See text for more detailed descriptions.

Figure 6A shows a false color image (Red: 0.63 μm; Green: 0.86 μm; Blue: 0.47 μm) for the AVIRIS scene over Ivanpah in California. The bright dry salt lake beds extending from top to bottom are seen obviously in the image. The green areas in the center left portion of the image are covered by vegetation. The dotted line in Figure 6B is the ATREM-derived surface reflectance spectrum over a soil pixel located at the center of the red box in Figure 6A. The curve is not spectrally smooth, particularly in the 0.86–1.20 μm range, due to residual errors in the ATREM atmospheric correction process. The solid line (vertically displaced for clarity) in Figure 6B is the smoothed spectrum. The spectrum in the 0.86–1.20 μm range becomes much smoother after the application of the cubic spline smoothing algorithm. By comparing the two curves in Figure 6B, it is also seen that major mineral absorption features centered near 2.20 and 2.34 μm are preserved after spectral smoothing, and no artificial absorption features in the entire 0.4−2.5 μm spectral range are introduced by the smoothing algorithm.

**Figure 7.**
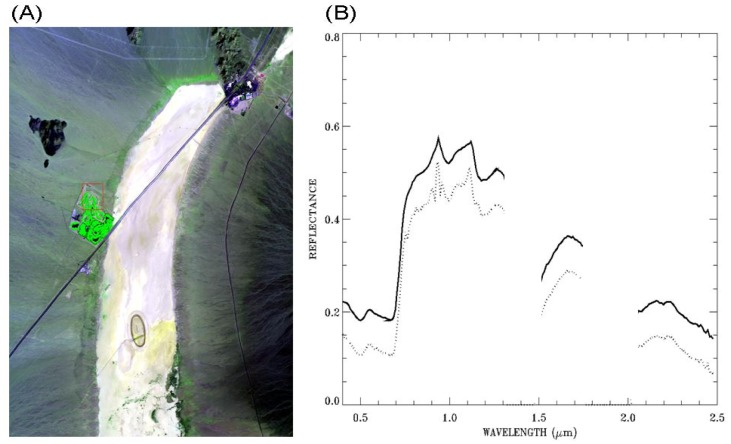
(**A**): a false color image (Red: 0.63 μm; Green: 0.86 μm; Blue: 0.47 μm) for an AVIRIS scene over Ivanpah in California, and (**B**): an ATREM-derived surface reflectance spectrum over a green vegetation pixel (dotted line) and the corresponding smoothed spectrum (solid line). See text for more detailed descriptions.

Figure 7 is similar to Figure 6, except for a spectrum over green vegetation covered area. The general shapes of the spectrum in the 0.5−2.5 μm range after smoothing (solid line in Figure 7B) are similar to those of green vegetation reflectance spectra measured in laboratories.

**Figure 8.**
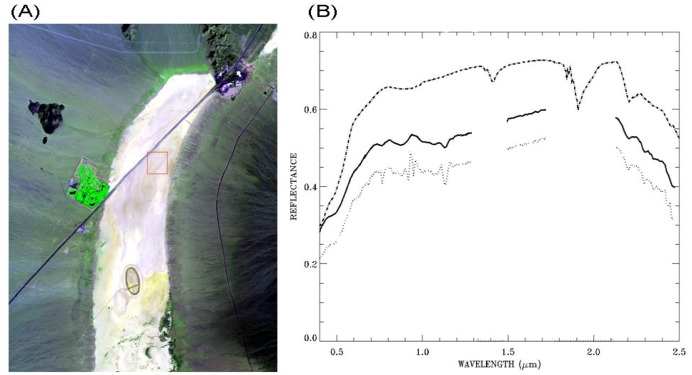
(**A**): a false color image (Red: 0.63 μm; Green: 0.86 μm; Blue: 0.47 μm) for an AVIRIS scene over Ivanpah in California, and (**B**): an ATREM-derived surface reflectance spectrum over an Ivanpah playa pixel (dotted line), the corresponding smoothed spectrum (solid line), and a field-measured spectrum (dash-dotted line). See text for more detailed descriptions.

Figure 8A is similar to Figure 6A. Figure 8B shows comparisons among ATREM-derived surface reflectance spectrum (dotted line) over an Ivanpah playa pixel located at the center of the red box in Figure 8A, the smoothed spectrum (solid line and vertically displaced by 0.075 in reflectance unit), and a field-measured spectrum (dash-dotted line and vertically displaced by 0.15 in reflectance unit). A major mineral feature centered at 2.2 μm is seen in all the spectra.

In order to quantify improvements in smoothness, first derivatives for all the spectra in the Ivanpah scene before and after application of the smoothing algorithm were calculated. After smoothing, the average absolute spectral derivative for all the bands, excluding those bands centered near 1.38- and 1.88-μm strong water vapor band absorption regions, over the scene decreased by 14%. The magnitude of the decrease is larger for a number of bands, including some bands located within the 0.94- and 1.14-μm water vapor band absorption regions where larger spectral residuals are observed (see Figure 7B). The scene-averaged spectral derivative for a band centered near 1.11 μm decreased by 20%. The decrease in spectral derivatives demonstrates the improvement in smoothness after applications of the cubic spline smoothing algorithm.

## 5. Discussion and Summary

During our development of the cubic spline smoothing technique for post-processing of surface reflectance spectra retrieved from AVIRIS data, we also tried another well-known filter, *i.e.*, the Savitzky-Golay (SG) filter [16]. The use of SG filter didn't produce satisfactory results. We observed that the peak positions of spectral features after application of the SG filter were not preserved. Therefore, we decided not to use the SG filter for our spectral smoothing purposes.

In summary, we have described a technique, which fits spectra “locally” in the spectral domain based on cubic spline smoothing, for quick post processing of apparent reflectance spectra derived from AVIRIS data using the ATREM code. Results from analysis of AVIRIS data acquired over Cuprite mining district in Nevada in June of 1995 and over Ivanpah in California in April of 2010 are presented. Very good agreement between our results and those of empirical line method in the 2.0–2.5 μm spectral region is obtained. It is expected that the use of ATREM code for retrieving surface reflectance spectra from AVIRIS data plus the application of additional spectral smoothing should yield high quality surface reflectance spectra comparable with those of reflectance spectra measured in laboratory conditions.
